# GLI1 orchestrates CXCR4/CXCR7 signaling to enhance migration and metastasis of breast cancer cells

**DOI:** 10.18632/oncotarget.5203

**Published:** 2015-09-16

**Authors:** Shingo Inaguma, Miho Riku, Hideaki Ito, Takumi Tsunoda, Hiroshi Ikeda, Kenji Kasai

**Affiliations:** ^1^ Department of Pathology, Aichi Medical University School of Medicine, Nagakute, Aichi 480-1195, Japan

**Keywords:** GLI1, CXCR4, CXCR7, migration, breast cancer

## Abstract

The up-regulation of chemokine receptors CXCR4 and CXCR7 impacts on the distant metastasis and prognosis of breast cancer, though knowledge about the regulatory mechanism of their expressions is limited. Meanwhile, the GLI transcription factors of Hedgehog signaling have been reported to play a pivotal role in the development and progression of many types of human cancer. In breast cancer, the increased expression of GLI1 correlated with metastasis and unfavorable overall prognosis, though its molecular mechanism is also not fully understood. Based on our findings that GLI1 enhanced the lung metastasis of breast cancer cells in a mouse model system, we comprehensively screened for genes up-regulated by GLI1 in breast cancer cells, and as such identified *CXCR4*, *CXCR7/ACKR3*, and actin-binding protein *LCP1/L-PLASTIN*, all of which have been reported to be involved in CXCL12-stimulating signaling. In breast cancer cells, we found that GLI1 and GLI2 up-regulated these expressions, while treatment with GLI-specific inhibitor GANT61 reduced the expressions. As for *CXCR4*, we confirmed it as a direct target of GLI1 through the reporter assay and the chromatin immunoprecipitation assay. We also found that GLI1 enhanced CXCL12-induced ERK phosphorylation and cell migration, both of which were blocked by either CXCR4-specific inhibitor or knockdown of *CXCR7* or *LCP1*. These evidences suggest an indispensable role of GLI1 in the migration and metastasis of breast cancer cells through CXCL12/CXCR4 signaling enhancement.

## INTRODUCTION

Breast cancer frequently exhibits distant metastasis to bone, lung, liver, and brain, which worsens morbidity and mortality associated with the disease. This tissue-specific metastasis has been explained in part by the expression of CXCR4 and its downstream signaling of breast cancer cells. CXCR4, a chemokine receptor that belongs to the superfamily of heptahelical G protein-coupled receptors (GPCRs), was thought to be an exclusive receptor for chemokine CXCL12 [1]. CXCL12 is highly secreted from the aforementioned metastatic target organs [2]. Consistently with this, the up-regulation of CXCR4 in breast cancer cells has been clinically correlated with the distant metastasis and unfavorable overall survival of breast cancer [3]. It has also been experimentally confirmed that the CXCR4 up-regulation enhanced the chemoattraction or directional migration of breast cancer cells to CXCL12 *in vitro* [2, 4], as well as the lung metastasis *in vivo* [2, 4–6]. However, to date, the molecular mechanism of CXCR4 expression has not been fully understood. While transcriptional regulators such as USF/c-myc [7], NFkB [8] and p53 [9] have been reported to contribute to the regulation of *CXCR4* expression, they are ubiquitously expressed; estrogen receptor-dependent up-regulation of CXCR4 in breast cancer cells has also been reported [10], but it does not account for the fact that a high level expression of CXCR4 predicts a poor prognosis for a “triple-negative” type of breast cancer, which does not express a hormone receptor [3].

Another chemokine receptor for CXCL12, CXCR7/ACKR3, was recently reported to play a crucial role for CXCR4-mediated metastasis. Upon CXCL12 stimulation, whereas CXCR4 evokes the activation of Gαi-mediated signaling of heterotrimeric G proteins, CXCR7 does not activate Gαi-mediated signaling, even when it binds to CXCL12: CXCR7 binds to CXCR4 and forms a heterodimer with it, and this CXCR4/CXCR7 heterodimer induces conformational rearrangement within CXCR4 and impairs CXCR4-mediated Gαi activation. Instead, CXCR4/CXCR7 heterodimer recruits β-arrestin and activates its downstream cascades, including MAPK/ERK pathway [1, 11–13]. Intriguingly, CXCR4 co-expressed with CXCR7 enhances more CXCL12-induced migration and lung metastasis of breast cancer cells than the sole expression of CXCR4 [13, 14]. However, despite the importance in the modulation of CXCR4-mediated signaling and cancer cell metastasis, the regulatory mechanism of CXCR7 expression has not been fully elucidated, either. In breast cancer cells, it was reported that, unlike CXCR4, the expression of CXCR7 was suppressed by estrogen receptor-mediated signaling [10, 15], leaving the question of how metastatic cancer cells up-regulate both CXCR4 and CXCR7 unanswered.

The zinc-finger transcription factors, GLI1, GLI2 and GLI3, are known as downstream effectors of Hedgehog signaling [16]. Among these, GLI1 and GLI2 have been thought to be crucial for the development and progression of many types of human cancers, including lung, pancreatic, prostate, and breast cancer [17]. Indeed, the expression of GLI1 is also associated with low survival rates of breast cancer patients [18]. At the molecular levels, GLI1 is indispensable for many aspects of cancer cell property in terms of the transcriptional regulation of downstream target genes, including *BHLHE41* for microsatellite instability [19], *ABCG2* for chemoresistance [20], *SNAI1* for epithelial-mesenchymal transition [21], *BCL2* for anti-apoptosis [22], and *BMI1* and *NANOG* for stemness [23–25]. These GLI1 target genes highlight a pivotal role of GLI1 in cancer biology, but whether and how GLI1 is linked to the metastasis of cancer is yet to be fully understood.

We here present evidence that GLI1 up-regulates the expression of *CXCR4, CXCR7* as well as *LCP1/L-PLASTIN*, an actin-binding protein crucial for CXCL12/CXCR4 signaling [26], in breast cancer cells. Consistent with our finding in a mouse model system that GLI1 enhanced the lung metastasis of mouse breast cancer cells, GLI1 enhanced CXCL12-induced migration of human breast cancer cells, and the migration was suppressed by any of CXCR4-specific inhibitor AMD3100 treatment, *CXCR7* knockdown, or *LCP1* knockdown. Concordantly, we found that GLI1 enhanced CXCL12-induced phosphorylation of ERK, which was mediated by CXCR4, CXCR7 and LCP1. These evidences indicated a role of GLI1 in enhancing the CXCL12/CXCR4/CXCR7 signaling axis, which may be responsible for tissue-specific metastasis of breast cancer cells.

## RESULTS

### GLI1 enhances metastatic potential of breast cancer cells

**Figure 1:**
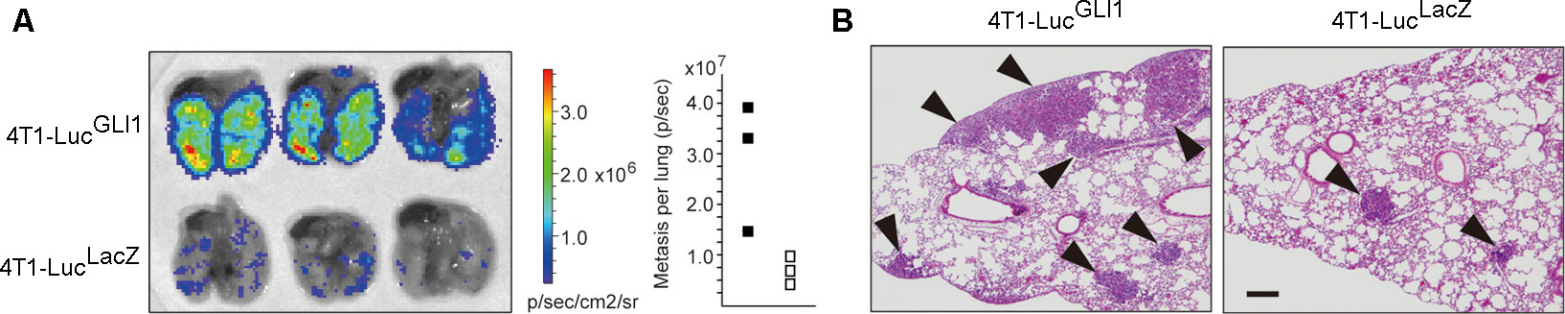
GLI activity enhances the lung metastasis of mouse breast cancer cells **A.** Bioluminescence image showing lung metastasis of 4T1-Luc^GLI1^ and 4T1-Luc^LacZ^ cells (*left*) and the number of metastatic foci (*right*). *Closed square*, 4T1-Luc^GLI1^: *open square*, 4T1-Luc^LacZ^. **B.** hematoxylin and eosin-stained lung cross-sections. *Arrows*, metastatic foci. *Bar*, 200 μM.

**Figure 2:**
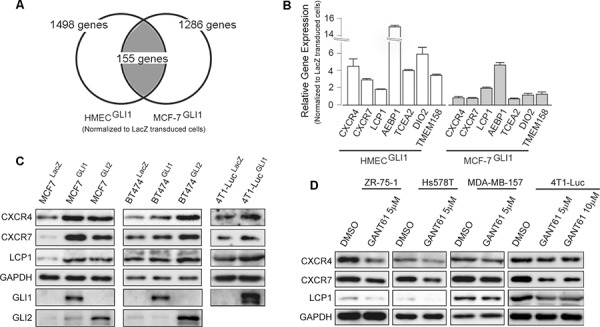
Screening of GLI1 target genes **A.** Schematic presentation of the experimental design of the microarray analysis. The list of 155 genes that were up-regulated by GLI1 is in Supplementary Table S1. **B.** validation by qRT-PCR analysis of the expression of GLI1 target genes. The result is represented as the fold increase (log2 scale) of expression in the GLI1-transduced cells relative to the LacZ-transduced cells of HMEC (*open columns*) and MCF-7 (*gray columns*). *Columns*, means of three independent experiments; *bars*, SD. **C.** Immunoblot analysis of MCF-7^LacZ^, MCF-7^GLI1^, MCF-7^GLI2^, BT474^LacZ^, BT474^GLI1^, BT474^GLI2^, 4T1-Luc^LacZ^ and 4T1-Luc^GLI1^. **D.** Immunoblot analysis of breast cancer cell lines treated with GANT61 or a vehicle (DMSO) for 48 hours.

The increased expression of GLI1 has been reported to clinically correlated with the metastasis and unfavorable overall prognosis of breast cancer [18], and yet its molecular mechanism has not been explained. To elucidate the role of GLI1 in breast cancer metastasis, we started with the experiments of lung metastasis using Balb/c mouse-derived breast cancer cells of 4T1-Luc, a derivative of 4T1 cells in which luciferase was stably transduced [5]. We lentivirally transduced either FLAG-tagged GLI1 or a control β-galactosidase (LacZ) into 4T1-Luc cells (4T1-Luc^GLI1^ and 4T1-Luc^LacZ^, respectively) and intravenously injected 5 × 10^5^ cells of those into Balb/c mice through a tail vein. Then days after injection, we removed the lung and examined its luciferase signal. We found that the GLI1 expression increased the number of metastatic foci of the lung (Figure 1A, 1B; see Figure 2C for GLI1 expression in 4T1-Luc^GLI1^), indicating the activity of GLI1 had to do with the metastatic potential of breast cancer cells. 4T1-Luc cells expressed a low amount but detectable levels of GLI1 (data not shown). To modulate endogenous activity of GLI1 in 4T1-Luc cells, we then treated 4T1-Luc cells for 48 hours with either GANT61, a specific inhibitor for GLI proteins that works by abolishing their DNA binding [27, 28], or a vehicle (DMSO) at 10 μM, and similarly injected these cells into Balb/c mice. We found that the treatment with GANT61 reduced the number of metastatic foci of the lung (Supplementary Figure S1; see Figure 2D for immunoblot analysis of GANT61-treated 4T1-Luc). These evidences experimentally indicated that the activity of GLI1 contributed to the increased metastatic potential of breast cancer cells.

### GLI1 up-regulates the expression of CXCR4, CXCR7 and LCP1

Given that GLI1 enhanced the lung metastasis of mouse 4T1-Luc cells, we assumed that GLI1 might up-regulate the expression of gene(s) responsible for the metastasis of human breast cancer cells. In order to first clarify downstream genes of GLI1 in human breast cancer cells, we employed a comprehensive screening assay: we lentivirally transduced either FLAG-tagged GLI1 or LacZ into MCF-7, a luminal A-type human breast cancer cell line, as well as primary culture cells of human mammary epithelium (HMEC). We then prepared cDNA probes from the transduced cells to serve for an Agilent 4×44k gene expression (cDNA) microarray analysis. We listed the genes showing more than a 2-fold up-regulation by GLI1 beyond LacZ transduction in both MCF-7 and HMEC (Figure 2A for the experimental design, Supplementary Table S1 for the list). The list contained 155 genes, and they included not only such known GLI1 targets as *PTCH1*, *ARC* [29], *BCL2* [22], and *BHLHE41* [19] but also *AEBP1*, *DIO2*, *TCEA2* and *TMEM189*, none of which were known as downstream genes of GLI1 (Figure 2B). In addition, we found that GLI1 up-regulated *CXCR4* expression in MCF-7 cells, as previously reported in medulloblastoma cells [30] and pancreatic cancer cells [31]. Intriguingly, we also found that GLI1 up-regulated the expression of *CXCR7* and *LCP1* (Figure 2B). Reportedly, CXCR7 modulates CXCR4 signaling and enhances CXCL12-induced cell migration and metastasis of breast cancer cells [13, 14], and LCP1 is implicated in the CXCL12-induced migration of chronic lymphocytic leukemia cells [26]. Assuming therefore that GLI1 up-regulates a set of these CXCL12-related signaling components in breast cancer cells, we focused our study on *CXCR4*, *CXCR7* and *LCP1*.

Lentivirally-transduced GLI1 and GLI2 were found to increase the expression of CXCR4, CXCR7 and LCP1 proteins in MCF-7 and in a luminal B-type BT474 as well as 4T1-Luc (Figure 2C). Conversely, treatment with GANT61 reduced those expressions in all tested breast cancer cell lines (Figure 2D). These evidences indicated that, in addition to *CXCR4*, the expression of *CXCR7* and *LCP1* was also in the downstream of GLI1 and GLI2 in breast cancer cells.

**Figure 3:**
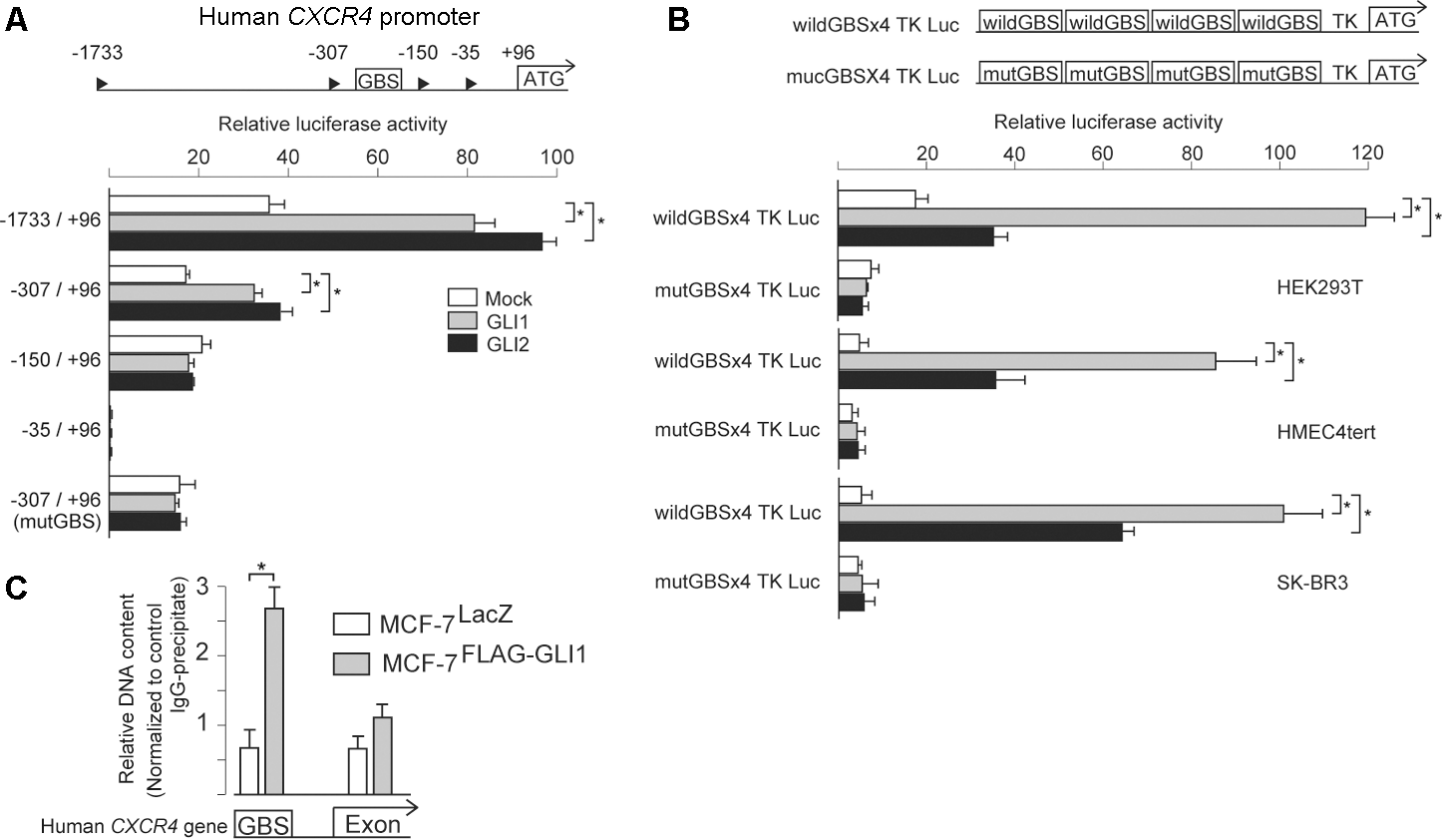
*CXCR4* is a direct target of GLI1 **A.** and **B.** Luciferase reporter assays. Cells were transiently transfected with the *firefly* luciferase reporter constructs harboring human *CXCR4* promoter fragments (A) or four tandem copies of either a wild-type or mutated GBS, flanked by the thymidine kinase minimum promoter (TKpro) (B) For the test of the GLI responsiveness, the expression vectors for either GLI1 (*gray columns*), GLI2 (*filled columns*) or a control vector (*open columns*) were co-transfected with *Renilla* luciferase expression vector. Reporter activity is represented as the fold activation relative to *Renilla* luciferase activity. *Columns*, means of three independent experiments; *bars*, SD; *, *P* < 0.01. **C.** Chromatin immunoprecipitation (ChIP) assay using MCF-7^LacZ^ (*open columns*) and MCF-7^GLI1^ *(gray columns*). Anti-DYKDDDDK (FLAG) antibody and an isotype-matched mouse IgG as a control were used for the immunoprecipitation. The result is represented as the fold increase of FLAG antibody-precipitated DNA relative to the control IgG-precipitated DNA. *Columns*, means of three independent experiments; *bars*, SD; *, *P* < 0.01.

Next, to confirm *CXCR4* regulation by GLI1, we analyzed the promoter region of the human *CXCR4* gene. Previously, Eberl *et al*. analyzed the regulatory region for the expression of *CXCR4* gene and predicted the GLI-binding site (GBS) in −217 to −179 (−217/−179) region from the transcriptional start site (+1) [31]. However, the luciferase reporter construct of *CXCR4* promoter harboring mutated GBS of −217/−179 was showed to respond to GLI1 yet, leaving the possibility of another GBS responding to GLI1 more efficiently. Therefore, to examine this, we cloned the 5′ flanking genomic region of 2kb or shorter length from the translation start site (ATG) (+96) of *CXCR4* gene [32] into the luciferase reporter vector pGL3-basic (Promega), and examined whether these constructs responded to either GLI1 or GLI2 in HEK293T cells (Figure 3A). We found that −1733/+96 and −307/+96, but not −150/+96 fragments, responded to both GLI1 and GLI2. As we found a putative GBS (5′-cgaccacccgc-3′) between nucleotide −181 to −171, which was nearby to −217/−179, we generated the −307/+96 luciferase reporter construct harboring a mutated GBS of −181/−171 (5′-aacttcttaac-3′) and found this mutated construct not to respond to either GLI1 or GLI2 (Figure 3A). To further confirm this GBS-dependent response of *CXCR4* promoter, we next generated artificial constructs containing four tandem copies of either a wild-type or mutated GBS, flanked by the thymidine kinase minimum promoter (TK pro), and examined whether these constructs responded to GLI1 and GLI2 in HEK293T cells, immortalized human mammary epithelial cells HMEC4*hTertp16shRNA* [33] (hereafter HMEC4tert), and HER2-type breast cancer cells SK-BR3. As expected, we found that the reporter construct containing a wild-type GBS, but not one containing a mutated GBS, responded to both GLI1 and GLI2 in these cell lines (Figure 3B). Finally, we performed a chromatin immunoprecipitation (ChIP) assay: we immunoprecipitated with anti-FLAG antibody from either MCF-7^GLI1^ or MCF-7^LacZ^ cells, extracted those DNA fragments, and served them as a template for qPCR analysis. We found that FLAG antibody precipitation enriched the GBS-containing fragment but not the exonic fragment, which was immunoprecipitated only from MCF-7^GLI1^ cells (Figure 3C). These evidences supported that *CXCR4* is also a direct target gene of GLI1 in breast cancer cells. As for *CXCR7* and *LCP1*, we analyzed their promoter region of up to 2kb length by the luciferase reporter assay and found that these reporter constructs did not respond to either GLI1 or GLI2, suggesting the possibility that *CXCR7* and *LCP1* would be indirect targets of GLI1 and GLI2 (data not shown).

### GLI1 enhances CXCL12-induced phosphorylation of ERK through CXCR4, CXCR7 and LCP1

**Figure 4:**
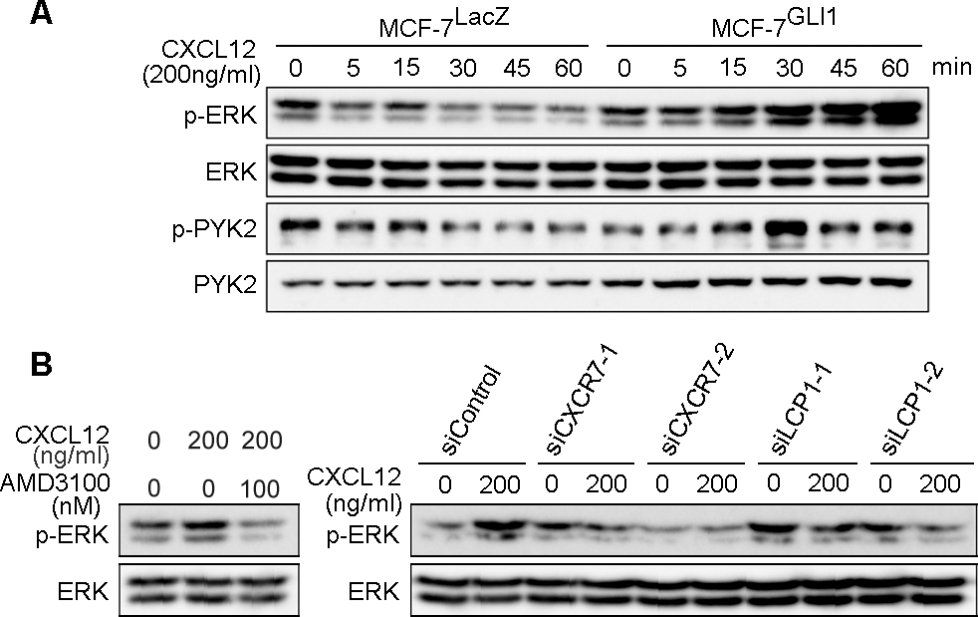
GLI1 enhances CXCL12-induced phosphorylation of ERK and PYK **A.** Immunoblot analysis of CXCL12-treated MCF-7^LacZ^ and MCF-7^GLI1^. **B.** Immunoblot analysis of AMD3100-treated or siRNA-transfected MCF-7^GLI1^ cells.

CXCR4 and CXCR7 belong to GPCR [1, 11]. CXCR4 activates intracellular signaling through a Gαi-mediated pathway of a heterotrimeric G protein upon the stimulation of CXCL12. But when CXCR7 is up-regulated, CXCR4 forms a heterodimer with CXCR7, and the CXCR4/CXCR7 heterodimer does not activate the Gαi signaling [12]; instead the heterodimer activates β-arrestin-dependent intracellular signaling that leads to the phosphorylation of downstream kinases including ERK1/2 [13]. Given that GLI1 up-regulated both CXCR4 and CXCR7 expressions, we examined whether GLI1 enhanced the CXCL12-induced phosphorylation of ERK1/2 and PYK2 kinases [34]. We treated MCF-7^LacZ^ and MCF-7^GLI1^ cells with CXCL12 and served their lysates for immunoblot analysis (Figure 4A). In MCF-7^LacZ^ cells, which harbored a weak expression of CXCR4 and CXCR7 (Figure 2C), CXCL12 treatment showed a subtle increase in phosphorylated ERK1/2 and PYK2 for a short duration. In MCF-7^GLI1^ cells, however, CXCL12 treatment showed a prominent and prolonged increase in them, indicating that GLI1 indeed enhanced the CXCL12-induced phosphorylation of these kinases (Figure 4A). Next, to confirm the involvement of CXCR4, CXCR7, and LCP1, we performed the following experiments: to analyze the CXCR4 involvement, we pre-treated MCF-7^GLI1^ cells with either AMD3100 or a vehicle and then treated them with CXCL12 for 60 minutes (Figure 4B, *left panel*); to analyze the involvement of CXCR7 and LCP1, we transfected MCF-7^GLI1^ cells with one of these siRNAs, *CXCR7*-specific (siCXCR7-1, 2), *LCP1*-specific (siLCP1-1, 2) or a control (siControl) siRNA, for 48 hours and then treated them with CXCL12 for 60 minutes (Figure 4B, *right panel*; see Supplementary Figure S2 for a validation of knockdown). As a result, we found that either CXCR4 inhibition or *CXCR7* knockdown (as well as *LCP1* knockdown) suppressed CXCL12-induced increase of phosphorylated ERK1/2 in MCF-7^GLI1^ cells. This observation indicated that GLI1 enhanced CXCL12-induced intracellular signaling downstream of the CXCR4/CXCR7 heterodimer. *LCP1* knockdown was also found to suppress the CXCL12-induced increase of phosphorylated ERK1/2 in MCF-7^GLI1^ cells (Figure 4B). However, we currently do not fully certain about its molecular mechanism; it might be possible that LCP1 modulates the intracellular sorting / distribution of the CXCR4/CXCR7 heterodimer or related signaling molecules to the cell surface, as reported in the case of another actin-binding protein α-actinin-1, which modulates the cell surface expression of G protein-coupled metabotropic glutamate receptor type 5b [35]. This possibility should be investigated in the future.

### GLI1 enhances CXCL12-mediated cell migration through CXCR4, CXCR7 and LCP1

**Figure 5:**
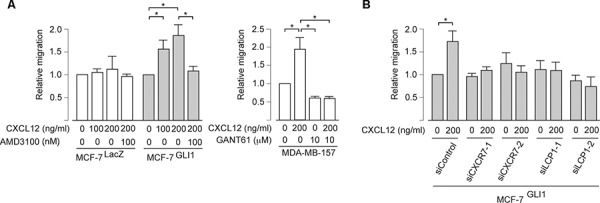
GLI1 enhances the CXCL12-induced migration through CXCR4, CXCR7 and LCP1 **A.** and **B.** Cell migration assay. Cells pretreated with either the indicated chemicals (A) or siRNAs (B) were resuspended in DMEM containing 5% bovine serum albumin and applied into the top chamber. CXCL12 was applied as an attractant in the bottom chamber. The effect of knockdown was validated by the immunoblot analysis (see Supplementary Figure S2). *Columns*, means of three independent experiments; *bars*, SD; *, *P* < 0.01.

**Figure 6:**
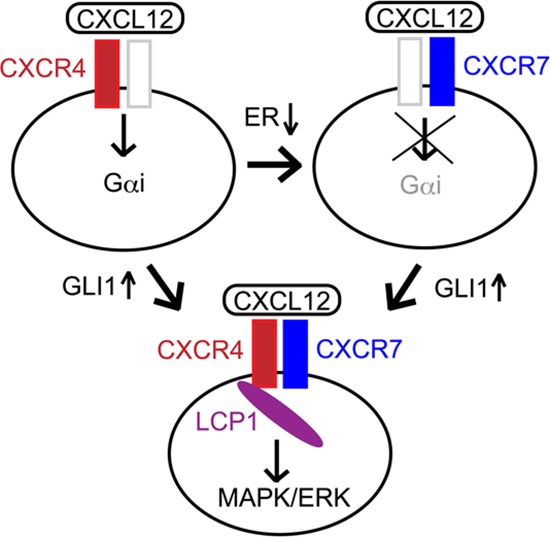
Proposed mechanism of the regulation of the CXCR4/CXCR7 signaling axis The activity of GLI1 up-regulates a set of the signaling molecules, CXCR4, CXCR7 and LCP1, leading to enhance CXCL12-induced MAPK/ERK pathway.

The up-regulation of CXCR4 is a key to enhancing the CXCL12-dependent migration of breast cancer cells [2, 4–6]. Indeed, we found that MCF-7^GLI^, but not MCF-7^LacZ^ cells, showed the enhancement of CXCL12-induced migration, while the migration was inhibited by AMD3100 in the Boyden chamber cell migration assay (Figure 5A, *left panel*). We also revealed that, while basal-type breast cancer cells MDA-MB-157 showed CXCL12-induced migration, this migration was blocked by pre-treatment with GANT61 (Figure 5A, *right panel*). These evidences indicated that GLI1 enhanced CXCL12-induced cell migration through CXCR4. In accordance with the above results, we found that transfection of *CXCR7*-specific siRNAs as well as *LCP1*-specific siRNAs reduced CXCL12-dependent migration in the Boyden chamber assay, confirming that the up-regulation of either CXCR7 or LCP1 contributed to the GLI1 enhancement of CXCL12-dependent migration (Figure 5B). Taken together, we propose the molecular mechanism of GLI1-mediated enhancement of the migration and lung metastasis of breast cancer cells as follows: as previous reported [10, 15], estrogen receptor-mediated signaling up-regulates the expression of CXCR4 but not CXCR7, leading to the CXCL12-induced activation of a Gαi-mediated pathway; in hormone receptor-negative cancer cells, down-regulation of estrogen receptor instead increases the expression of CXCR7, which does not activate Gαi-mediated pathway; however, in the situation of GLI1 activation, CXCR4, CXCR7 and LCP1 are collectively up-regulated and evoke the enhancement of the CXCL12-stimulating MAKP/ERK pathway, which leads the enhanced migration and metastasis of breast cancer cells (Figure 6).

## DISCUSSION

CXCR4 has been reported as a prognostic biomarker in various type of cancer, including gastric cancer [36], colorectal cancer [37], esophageal cancer [38], pancreatic cancer [39], leukemia/lymphoma [40, 41], ovarian cancer [42] as well as breast cancer [3]. The expression of CXCR7 has also been revealed to be up-regulated in these cancers [11]. However, how these two key molecules are up-regulated in cancer cells has not been fully elucidated.

In the present study, we showed that the activity of GLI1 was implicated in the highly metastatic potential of 4T1-Luc cells *in vivo*. Furthermore, we revealed that GLI1 up-regulated the expression of CXCR4, CXCR7, and LCP1 to enhance the CXCL12-induced ERK phosphorylation and migration of breast cancer cells. These evidences provide insight into the molecular mechanism that explains why GLI1 expression and CXCR4/CXCR7 axis are both linked to a poor prognosis of breast cancer patients. Indeed, the expression of GLI1 has been shown to be involved in the progression of the aforementioned types of cancer [18, 19, 43–47], suggesting therefore the possibility that GLI1 might be a therapeutic target to control the distant metastasis of not only breast cancer but also the other types of cancers.

## MATERIALS AND METHODS

### Cells

Human breast cancer cell lines were purchased from the ATCC. Primary culture of normal human mammary gland duct epithelium (HMEC) was purchased from LONZA (Switzerland). Immortalized normal human mammary gland duct epithelium (HMEC4*htertshp16*) was kindly gifted from Dr. Tohru Kiyono (the National Cancer Center Research Institute, Japan) and Dr. Denis Galloway (Fred Hutchinson Cancer Research Center).

### Microarray analysis

Total RNA was purified from cells for cDNA microarray analysis using Agilent 4×44K cDNA microarrays (Agilent Technologies) as previously described [19]. The microarray data are available online *via* the Gene Expression Omnibus (GEO) under the accession numbers GSE64350.

### Plasmid and lentiviral vectors

Expression vectors of FLAG-tagged human GLI1 and GLI2 were constructed from pCMVTNT (Promega). Luciferase reporter vectors containing human *CXCR4* promoter were constructed from the luciferase reporter pGL3 (Promega). Luciferase reporter vectors, wildGBSx4TKLuc and mutGBSx4TKLuc, which harbor four copies of the wild-type or mutated GLI-binding site within the human *CXCR4* promoter, were also from pGL3 (Promega). Lentiviral vectors expressing either human GLI1 or LacZ were constructed from the CSII-CMV-MCS-IRES2-Bsd plasmid, which was kindly provided by Dr. Hiroyuki Miyoshi (RIKEN BioResource Center, Japan).

### Luciferase reporter assay and qRT-PCR

Luciferase reporter assays were conducted using the Dual-Glo luciferase system co-transfected with a control *Renilla* luciferase expression vector (Promega) as previously reported [48]. Quantitative RT-PCR (qRT-PCR) was performed using a StepOnePlus™ real-time PCR system (Applied Biosystems) in conjunction with probes for TaqMan Gene Expression Assays (Applied Biosystems) according to the manufacturer's protocol.

### Chromatin immunoprecipitation (ChIP) assay

ChIP assay was done as previously described [19]. Anti-DYKDDDDK (FLAG) antibody (clone 2H8, TransGenic, Japan) and an isotype-matched mouse IgG as a control were used for the immunoprecipitation. For the quantitative of precipitated DNA, StepOne™ system was used with customized qPCR primers (Applied Biosystems, USA) as follows: for a fragment containing GBS of the *CXCR4* promoter, 5′-aacattccagagcgtgtagtgaa-3′, 5′-ccacgggaatggagagattatctatg-3′ and 5′-cacgtaaagctagaa-atgat-3′ ; for a fragment of the exon, 5′-cgaccacccgcaaacag-3′, 5′-gcagacgcgaggaagga-3′ and 5′-caagccgcgcacctc-3′. The result is represented as the fold increase of FLAG antibody-precipitated DNA relative to the control IgG-precipitated DNA.

### siRNAs and antibodies

The following 21-nucleotide duplex siRNAs against human *CXCR7* (siCXCR7-1, siCXCR7-2), *LCP1* (siLCP1-1, siLCP1-2) as well as a control (siControl), were synthesized: siCXCR7-1, 5′-ccuucauuuacauuuucaudTdT-3′, 5′-augaaaauguaaaugaaggdTdT-3′; siCXCR7-2, 5′-ccuuauaa-auguauuugaadTdT-3′, 5′-uucaaauacauuuauaaggdTdT-3′; siLCP1-1, 5′-gcuuugaugaguuuaucaadTdT-3′, 5′-uugauaaacucaucaaagcdTdT-3′; siLCP1-2, 5′-gaacaaucaacaaaaagaadTdT-3′, 5′-uucuuuuuguugauuguucdTdT-3′; siControl, 5′-cguacgcggaauacaacgadTdT-3′, 5′-ucguuguauuccgcguacgdTdT-3′. Used antibodies were as follows: anti-CXCR4 (Abcam, USA), anti-CXCR7 (Abcam, USA), anti-LCP1 (GeneTex, USA), anti-GLI1 (Novus), anti-GLI2 (Santa Cruz Biotechnology), anti-GAPDH (Santa Cruz Biotechnology, USA), anti-ERK1/2, anti-phosphorylated ERK1/2, anti-PYK2 and anti-phosphorylated PYK2 (Cell Signaling).

### Migration assay and *in vivo* lung metastasis assay

Cell migration assay using the Boyden-Chamber (BD Biosciences, USA) as previously described [49]. Briefly, cells pretreated with either AMD3100 (SIGMA, USA) or a vehicle (DMSO) were resuspended in DMEM containing 5% bovine serum albumin and applied into the top chamber. CXCL12 (R&Dsystems, USA) was applied as an attractant in the bottom chamber. The *in vivo* lung metastasis assay was carried out as follows: 5 × 10^5^ cells of either MCF-7^GLI1^ or MCF-7^LacZ^, or 10^6^ cells pre-treated with either 10 μM GANT61 or a vehicle (DMSO) for 48 hours were intravenously injected into 8-week-old female Balb/C mice; ten days after injection, the lung was harvested and its bioluminescence was monitored by the IVIS system (Caliper LifeScience, USA); after taking the images, the lung was fixed in formalin and served for a tissue-section. All animal procedures were approved by the animal ethics committee of Aichi Medical University.

## SUPPLEMENTARY FIGURES AND TABLE


