# Supplementary material for: GLI1 orchestrates CXCR4/CXCR7 signaling to enhance migration and metastasis of breast cancer cells
Source: Oncotarget. 2015 Sep 16;6(32):33648–57. doi: 10.18632/oncotarget.5203 (PMC4741792; doi:10.18632/oncotarget.5203)
Supplement: Supplementary file 1 [file oncotarget-06-33648-s001.pdf]

SUPPLEMENTARY FIGURES AND TABLE

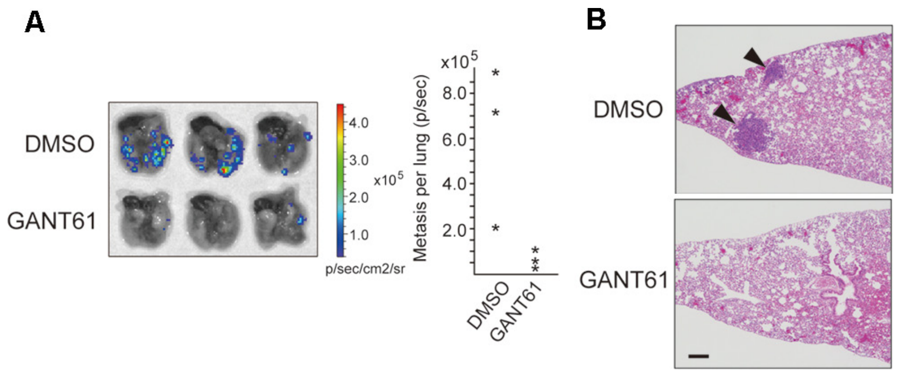

Supplementary Figure S1: GANT61-treatment reduced the lung metastatic foci of 4T1-Luc mouse breast cancer cells. A. Bioluminescence image showing lung metastasis of 4T1-Luc cells (*left*) and the number of metastatic foci (*right*). 10^6 cells pre-treated with either 10 µM GANT61 or a vehicle (DMSO) for 48 hours were intravenously injected into 8-week old female Balb/C mice. Ten days after injection, the lung was harvested and its bioluminescence was monitored by the IVIS system (Caliper LifeScience, USA). After taking the images, the lung was fixed in formalin and served for a tissue section. All animal procedures were approved by the animal ethics committee of Aichi Medical University. **B.** Hematoxylin and eosine-stained lung cross sections. *Arrows*, metastatic foci, *Bar*, 200 µM.

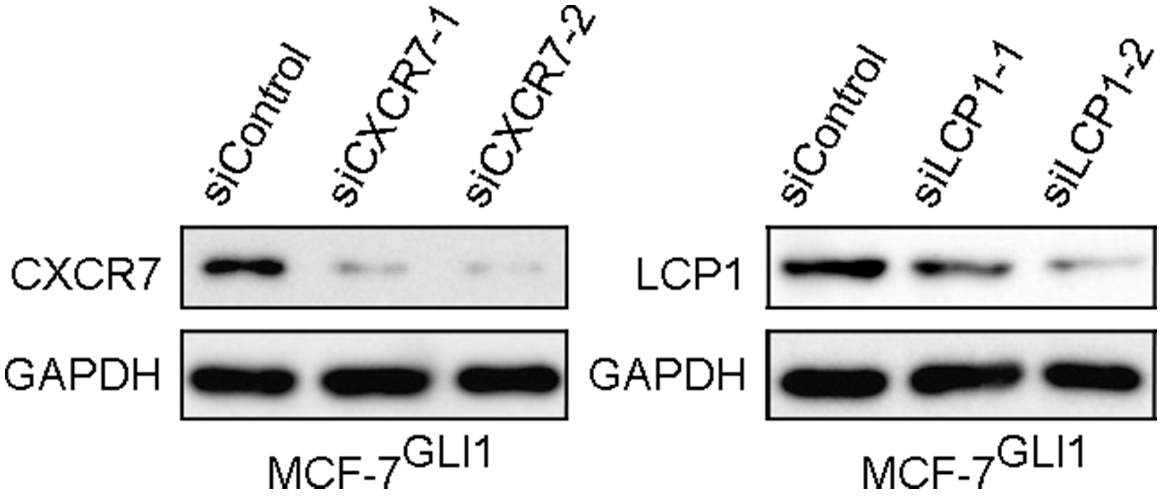

Supplementary Figure S2: siRNA validation. MCF-7^{GLI1} cells were transiently transfected with the indicated siRNA for 48 hours and served for immunoblot analysis.

Supplementary Table S1: Up-regulated genes by GLI1

ACKR3	AEBP1	AGXT	AMTN	APOE	ARC	ARHGAP26	ASS1
BCL2	BCOR	BHLHE41	BIN1	BMP8B	BOK-AS1	BST2	C11orf96
C2orf54	CABP1	CCNA1	CD177	CDRT1	CMPK2	CNIH3	COL20A1
COL4A2	CPZ	CRCT1	CRIP1	CXCR4	CYP26A1	CYP4B1	DIO2
DLEC1	DLX5	DNAJB8	DPYD	DRD2	EDN2	ENPP2	EPHA4
FAM129A	FAM213A	FAM221B	FAXC	FBLN2	FBN2	FGD3	FLJ43681
FOXQ1	FOXS1	FREM1	FST	FYB	GAS6-AS1	GCGR	GLI1
GSTA4	HCAR1	HEYL	HHIP	HIVEP3	HLA-B	HLA-DPB1	IL1R2
ITGB2	KALRN	KIAA0195	KLF2	KRT42P	KRTAP9-3	KRTAP9-4	KRTAP9-8
KRTAP9–9	KTN1-AS1	LAMC3	LCP1	LILRA2	LILRA3	LILRS4	LILRB1
LILRB2	LILRB3	LIMS2	LINGO1	LMCD1	LMO7	LOC158434	LY6D
LYNX1	LYPD1	MAN1C1	MFSD4	MGAT3	MMP11	NPR3	NPTX1
NPTX2	NRP1	NTN1	NTNG2	OBSCN	OXTR	PDCD1	PHGDH
PKDCC	PLA2G4D	PLEKHA4	PLEKHG4	PNMA6C	PNPLA3	PPP2R2B	PRB1
PRB2	PRB3	PRB4	PRKCQ	PRODH	PSAPL1	RASA3	RBFOX3
RENBP	RGMA	RIMKLA	S1PR3	SBSPON	SCARNA9	SECTM1	SERPINA5
SHANK2	SLC15A3	SLC1A3	SLC1A7	SLC2A14	SLC7A8	SOCS2-AS1	SOHLH1
SOSTDC1	SSPN	STOM	SUSD2	SYNPO	SYTL1	TCEA2	TENM2
TEX101	TFF3	TMEM158	TNNC1	TRIM49	UCA1	WNT5A	XIST
XLOC_010517	XLOC_014288	XLOC_014512					
